# Role of Cardiac Natriuretic Peptides in Heart Structure and Function

**DOI:** 10.3390/ijms232214415

**Published:** 2022-11-20

**Authors:** Riccardo Sarzani, Massimiliano Allevi, Chiara Di Pentima, Paola Schiavi, Francesco Spannella, Federico Giulietti

**Affiliations:** 1 Internal Medicine and Geriatrics, Istituto di Ricovero e Cura a Carattere Scientifico-Istituto Nazionale di Ricovero e Cura per Anziani (IRCCS INRCA), 60127 Ancona, Italy; 2 Department of Clinical and Molecular Sciences, Università Politecnica delle Marche, 60126 Ancona, Italy

**Keywords:** natriuretic peptides, ANP, BNP, CNP, osteocrin, musclin, heart, heart failure, review

## Abstract

Cardiac natriuretic peptides (NPs), atrial NP (ANP) and B-type NP (BNP) are true hormones produced and released by cardiomyocytes, exerting several systemic effects. Together with C-type NP (CNP), mainly expressed by endothelial cells, they also exert several paracrine and autocrine activities on the heart itself, contributing to cardiovascular (CV) health. In addition to their natriuretic, vasorelaxant, metabolic and antiproliferative systemic properties, NPs prevent cardiac hypertrophy, fibrosis, arrhythmias and cardiomyopathies, counteracting the development and progression of heart failure (HF). Moreover, recent studies revealed that a protein structurally similar to NPs mainly produced by skeletal muscles and osteoblasts called musclin/osteocrin is able to interact with the NPs clearance receptor, attenuating cardiac dysfunction and myocardial fibrosis and promoting heart protection during pathological overload. This narrative review is focused on the direct activities of this molecule family on the heart, reporting both experimental and human studies that are clinically relevant for physicians.

## 1. Introduction

Diseases of the heart and cardiovascular (CV) system are the main cause of disability and death worldwide [1]. Several CV risk factors and conditions, such as hypertension, dyslipidemia, obesity and insulin resistance, lead to multiple organ damage. Together with the involvement of the small and large arterial vessels, these risk factors and conditions affect the heart through increased heart afterload, perivascular myocardial fibrosis, left ventricular remodeling and hypertrophy (LVH), myocardial ischemia and necrosis, leading to heart failure (HF) [2,3]. How can the heart defend itself from these common causative factors of myocardial damage?

In recent decades, evidence from multiple research groups has emphasized the crucial protective role of the natriuretic peptides (NPs) expressed by the heart [4,5,6,7,8,9]. Atrial NP (ANP) and brain (or B-type) NP (BNP) are true hormones produced and released by cardiomyocytes, exerting pleiotropic systemic effects that range from blood pressure (BP) regulation to both glucose and lipid metabolism, with a wide spectrum of cardio-metabolic properties, including vasodilation, natriuresis and inhibition of the renin–angiotensin–aldosterone system (RAAS), as well as lipid mobilization and oxidation, adipocyte browning and improved insulin sensitivity [10,11,12]. On the other hand, they also act locally on the heart, exerting both paracrine and autocrine activities, mainly preventing hypertrophy, fibrosis, arrhythmias and cardiomyopathies, counteracting the development and progression of HF [9,13,14,15]. This narrative review is focused on the direct activities of NPs on the heart itself, reporting both experimental and human studies that are clinically relevant for physicians. Particular emphasis is placed on HF-related aspects and novel emerging data concerning the crosstalk between the NPs system and musclin/osteocrin, a secretory NP-like peptide implicated in the maintenance of CV health.

## 2. Cardiac Natriuretic Peptides, Their Receptors and Metabolism: A Long History Made Short

Cardiac NPs are synthesized as precursor proteins (inactive preprohormones), undergo intracellular modification to prohormones (pro-ANP and pro-BNP) and are subsequently cleaved in their active forms [16,17]. Pro-ANP is mainly expressed by atrial tissue under physiological conditions, whereas in the presence of HF, it is also expressed by ventricle tissue. Pro-ANP is stored in secretory granules, mainly of cardiomyocytes, and cleaved into the 28 amino acid biologically active hormone (ANP) and the 98 amino acid N-terminal fragment (NT-proANP) by corin, a transmembrane serine protease, whose loss of function leads to a disease phenotype characterized by high BP with reduced ANP activity, in addition to contributing to the pathogenesis of HF [18,19]. Pro-BNP is synthesized mainly by ventricular myocytes; instead of being stored, it is produced and secreted in bursts. Whereas activation of pro-ANP occurs on the cell surface during secretion, pro-BNP is cleaved into the 32 amino acid active hormone (BNP) and the 76 amino acid N-terminal fragment (NT-proBNP) inside the cells by furin, an intracellular serine endopeptidase, and secreted in cleaved forms [20,21]. The expression and release of both ANP and BNP occurs in response to wall hemodynamic stress resulting from increased extracellular volume and cardiac transmural pressure in a context of augmented cardiac mechanical stress, such as in HF or myocardial ischemia [22]. Moreover, ANP is also released in response to elevated concentrations of sodium [23].

In addition to cardiac NPs (ANP and BNP), a third actor exists, the C-type natriuretic peptide (CNP), which has no natriuretic properties and is mostly produced by vascular cells [24]. There are three subtypes of NPs receptors: NP receptor A (NPR-A), also called GC-A; NP receptor B (NPR-B), also called GC-B; and NP receptor C (NPR-C). Both NPR-A and NPR-B are transmembrane receptors with guanylyl cyclase (GC) activity; NPR-A is the principal receptor for ANP and BNP, whereas NPR-B has high affinity for CNP [25]. Binding of ANP and BNP to NPR-A induces intracellular generation of the second messenger cyclic guanosine monophosphate (cGMP) which, in its downstream cascade, activates multiple targets, including cGMP-dependent protein kinases (PKGs), cGMP-gated ion channels and cGMP-regulated cyclic nucleotide phosphodiesterases. These mediators account most of the biological effects of NPs [8]. NPR-A is widely expressed in many tissues, especially in the vasculature, heart, adipose tissue, kidneys, lungs, adrenal glands, brain and liver [26,27], whereas NPR-B is expressed mostly in bone and fibroblasts [8]. The third receptor, NPR-C, exhibits no GC activity, resulting mainly a clearance receptor for cardiac NPs by incorporating them into cells for subsequent lysosomal breakdown [28]. NPR-C is also able to couple with inhibitory G proteins (Gi), causing inhibition of adenylyl cyclase and activation of phospholipase-C, providing some direct signaling functions that might contribute to effects in cardiac tissue [29,30]. NPR-C is highly expressed in endothelial cells [31], in addition to adipose tissue and kidneys, and binds all NPs, although the binding affinity is higher for ANP and CNP than for BNP, resulting in a longer plasma half-life of BNP compared to ANP [32]. Cardiac NPs are also silenced as a result of enzymatic degradation by neprilysin (NEP), a ubiquitous zinc-dependent membrane metalloendopeptidase that is expressed mainly in smooth muscle cells, endothelial cells, cardiac myocytes, fibroblasts and kidneys [33].

## 3. ANP and BNP: Peptides Released by the Heart for the Benefit of the Heart

### 3.1. Antihypertrophic Effects of ANP and BNP on the Myocardium

The evidence of a link between NPs and cardiac hypertrophy starts with mouse models with genetic inactivation of ANP, BNP or NPR-A [34,35,36,37,38]. These murine models showed an overall increase in BP [37,38,39]. In pro-ANP knockout mice, hypertension and cardiac hypertrophy developed proportionally to the ANP decrease and to the dietary sodium chloride increase [38,39]. On the contrary, pro-BNP knockout mouse models mainly developed heart fibrosis but not hypertension or heart hypertrophy [40], suggesting that BNP at physiological concentrations is less clearly involved in BP regulation and fluid–electrolyte balance compared to ANP. However, overexpression of BNP gene with a subsequent increase in plasma BNP levels reduced BP in generated transgenic mice [41], suggesting that BNP affects BP regulation at higher concentrations, such as in HF. In Dahl salt-sensitive rats, BNP-knockout lines demonstrated adult-onset hypertension compared with age-matched controls. Moreover, increased left ventricular mass with LVH was observed in young adult knockout rats, apparently before frank hypertension ensued, although part of the cardiac damage was still mediated by increased BP, as evidenced by the simultaneous progressive nephropathy with proteinuria, fibrosis and glomerular alterations [37]. An analysis of the strongest differentially expressed pathways in knockout mice demonstrated clear trends in enhanced contractility and increased calcium (Ca2+) influx, together with an altered expression of cardiac repair, regeneration, contractility and cyclic adenosine monophosphate (cAMP) pathways. In another mouse model in which NPR-A was selectively abolished in cardiomyocytes only, increased afterload (by aortic banding) led to both LVH and fibrosis and was associated with increased death, demonstrating relevant paracrine (and hormonal) activity of NPs on cardiomyocytes [42]. In a murine model of pregnancy and lactation with a general genetic deletion of NPR-A, severe cardiac hypertrophy accompanied by fibrosis and left ventricular dysfunction developed in the lactation period, similar to a peripartum cardiomyopathy-like remodeling [43]. Moreover, this phenomenon was associated with increased expression of interleukin-6 and an inflammatory pathway similar to that observed in postpartum myocarditis. Interestingly, in a murine model of human obesity-related HF with preserved ejection fraction (HFpEF), it was observed that NPR-C plays a pivotal role also in the development of right ventricular dysfunction and hypertrophy [44].

Similar evidence has been reported in human clinical studies. In young normotensive subjects, specific NPR-A gene variants are associated with family history of hypertension and early diastolic dysfunction (prolonged isovolumetric time) [45]. Rubattu et al. showed that one promoter variant in the ANP gene and one in the NPR-A gene were associated with LVH in a cohort of hypertensive patients, apparently regardless of BP levels [46]. Moreover, the single nucleotide polymorphism (SNP) rs5068 located in the three prime untranslated region (3′-UTR) of the ANP gene has a minor G allele associated with increased circulating levels of ANP, leading to reduced BP, reduced odds of hypertension and a favorable cardiometabolic profile, such as lower prevalence of obesity, type 2 diabetes and metabolic syndrome, all conditions favoring cardiac hypertrophy [47,48,49,50]. In non-diabetic subjects, the rs5068 minor G allele was found to be associated with a reduced prevalence of LVH [51].

Although it is difficult to separate the direct antihypertrophic effects of NPs from the indirect effects mediated by an improved BP and cardiometabolic profile, the favorable role of cardiac NPs in the genesis/progression of myocardial hypertrophy is widely evident and promising in terms of future therapeutic opportunities. Currently, sacubitril/valsartan, a combined neprilysin inhibitor and angiotensin II (AngII) AT1 receptor antagonist (ARNI), showed beneficial effects in HF, leading to a reduction in hospitalizations and mortality, especially in HF with reduced ejection fraction (HFrEF) [52]. It increases NPs circulating levels, mainly ANP rather than BNP [53], through inhibition of neprilysin activity, and contributes both to the prevention/reversal of left ventricular hypertrophy and to counteraction of pulmonary hypertension and right ventricular remodeling [54,55].

### 3.2. Antifibrotic Effects of ANP and BNP on the Myocardium

Myocardial perivascular fibrosis is the hallmark of pathological heart changes in hypertension [56,57]. Perivascular and interstitial myocardial fibrosis lead to increased myocardial stiffness and cardiac dysfunction, commonly evolving in HFpEF and HFrEF [58]. Strong evidence suggests that the renin–angiotensin–aldosterone system (RAAS), the transforming growth factor beta 1 (TGF-β1) and endothelin-1 (ET-1) are important mediators of cardiac fibrosis [59,60]. Both ANP and BNP have been found to inhibit AngII- and norepinephrine-induced proliferation of cardiac fibroblasts [61,62]. The NP/cGMP/PKG pathway exerts its antifibrotic effect by antagonizing signaling, thus contrasting the pro-fibrotic actions of TGF-β1, AngII and ET-1 in cardiac myocytes and fibroblasts [63]. Some studies have reported a marked reduction in TGF-β1-induced selective atrial fibrosis in NPR-C knockout mice compared with the wild type, likely as a result of the indirect amplification of NPR-A signaling [64]. The atrial expression of NPR-C is up to 17-fold higher than ventricle expression, and this abundance of NPR-C in the atria could, in part, explain the differential response to TGF-β1-stimulated fibrosis between the atria and the ventricles [64]. Inversely, other experimental evidence obtained using different methodologies showed that the deletion of NPR-C in mice leads to basal atrial fibrosis and cardiac arrhythmias, as well as exacerbated AngII-induced atrial fibrosis [65,66], likely mediated by the less studied ability of NPR-C to activate inhibitory G proteins. In this context, although further studies are needed to understand the interaction between NPR-C and atrial remodeling in humans, the first mechanism appears to be the most supported by clinical evidence. In most animal models in which ANP/BNP effects mediated by NPR-A are hampered, increased BP, myocardial hypertrophy, fibrosis and ventricular dysfunction are common findings. However, the two cardiac NPs (ANP and BNP) may have a different weight in cardiac remodeling. Animal models lacking ANP showed mainly salt-dependent hypertension and heart hypertrophy, whereas fibrosis and ventricular dysfunction were reported to a lesser degree [38]. On the other hand, myocardial fibrosis appeared prominent in a mouse model with general ablation of the BNP gene. The hearts of these mice had similar gross anatomy, and heart-to-body weight ratios were not significantly different from those of control mice [40]. In older mice lacking BNP, several subendocardial focal fibrosis lesions were prominent, and an exaggerated cardiac fibrosis was documented in response to ventricular pressure overload, together with increased angiotensin-converting enzyme (ACE) and TGF-β3 messenger RNA (mRNA) expression, suggesting that BNP plays a critical role in preventing the development of cardiac fibrosis [67]. Therefore, it is supposed that ANP and BNP might play complementary roles in the regulation of cardiac remodeling; ANP may act mainly as an antihypertensive and antihypervolemic factor via NPR-A expressed in blood vessels and kidneys [68], whereas BNP, predominantly released by ventricles in response to ventricular overload/stretch, may act as an autocrine/paracrine antifibrotic factor through NPR-A expressed by ventricles [69]. NPs also act as hormones and mediate antifibrotic action in the cardio–renal axis through systemic release. In transgenic mice overexpressing BNP in the liver and subjected to subtotal nephrectomy, glomerular injury and interstitial fibrosis in the remnant kidney were significantly prevented [70]. In the clinical setting, this could explain, at least in part, the systemic beneficial effects of sacubitril/valsartan on the cardio–renal axis, exerting a protective role on the kidney and reducing risk of worsening renal function, as shown in recent clinical studies and meta-analyses [71,72].

### 3.3. Antiarrhythmic Effects of ANP and BNP

Cardiac NPs exert antiarrhythmic effects, both directly and indirectly (i.e., by antagonizing fibrosis). Atrial fibrillation (AF) is the most common cardiac arrhythmia encountered in clinical practice [73], especially in older patients [74]. Although there are different clinical forms of AF with respect to time and reversibility, atrial remodeling is the substrate for AF in most cases [73], as documented in both animal models [75,76,77] and human studies [78]. Atrial enlargement with structural remodeling and fibrosis, extracellular matrix expansion between bundles of myocytes and inflammatory cells infiltration [79] impair intermyocyte coupling, interfering with electrical continuity, representing well-established factors of the pathophysiology of AF. In this setting, BNP exerts both antifibrotic effects on cardiac fibroblasts and anti-inflammatory effects on monocytes, attenuating monocyte chemotaxis by activation of NPR-A, as previously reported [80]. In Dahl salt-sensitive rats with genetic BNP deletion, a significant elongation of the QT interval was detected after 9 months of prolonged hypertension and increased cardiac fibrosis and stiffness [37].

Moreover, BNP levels are predictors of AF onset and progression, as observed in several studies [81,82,83,84]. In the St Vincent’s STOP-HF (Screening To Prevent Heart Failure) study, elevated peripheral blood BNP levels were found to be helpful in identifying patients at risk of AF [85]. BNP has also been demonstrated to be increasingly expressed in human right atrial tissue as fibrosis and profibrotic macrophages increase [86], and myocardial BNP expression is significantly correlated with circulating BNP levels. In addition to AF, high BNP plasma levels have been found to predict sudden cardiac death and ventricular arrhythmias in patients with ischemic heart disease and left ventricular dysfunction [87]. In the Atherosclerosis Risk in Communities (ARIC) study, longitudinal changes in NT-proBNP plasma levels were associated with an increased atrial and ventricular arrhythmia burden [88]. These associations are consistent with the evidence that ventricular stretch, the primary stimulus for BNP secretion, results in proarrhythmic electrophysiological changes, potentially leading to ventricular arrhythmias [89].

There is a close relationship between ANP and arrhythmias. ANP seems to play a crucial role in differentiation of cardiac progenitor cells and cardiomyocytes. In murine models, ANP is expressed in the primitive heart tube by E8.5 (embryonic day 8.5) and subsequently downregulated in the murine ventricular chambers [90]. However, ANP expression persists after the E15 stage in some ventricular cells that are destined for the ventricular conduction system [91]. The ANP/NPR-A system is also involved in the full development of Purkinje fibers [92]. ANP gene variants are clearly associated with AF and have been documented to be a human genetic cause of familial AF in otherwise healthy myocardium. In 2008, in a large family of European descent in which familial AF segregated as an autosomal dominant trait, a novel two-base-pair deletion with frameshift mutation in the pro-ANP gene was identified [93]. Circulating levels of the mutant (Mut)-ANP were 5–10 fold higher in affected family members compared with wild-type (WT)-ANP, in part due to increased resistance to proteolytic degradation [94]. The relationship between ANP and AF is complex, requiring further studies to be elucidated. For example, a predictive score based on NT-proANP plasma levels showed an association with the presence of low-voltage areas (LVAs), which indicate an advanced disease stage in AF with dilated left atria [95]. Prediction of LVAs before catheter ablation could affect both the prognosis and therapeutic management in AF patients, as they are associated with worse outcomes after pulmonary vein isolation [96]. All these data show that NPs also affect cardiac rhythm and the development of both atrial and ventricular arrhythmias.

### 3.4. Cardiometabolic Effects of ANP and BNP

Cardiac NPs exert a wide spectrum of cardiometabolic actions, leading to more favorable glycemic and lipid profiles [11,12]. Among others, these actions include inhibition of cholesterol biosynthesis stimulated by AngII [97], promotion of the browning of white adipocytes [84], increased triglyceride degradation [98] and glucose uptake in adipocytes [99]. All these effects collectively improve insulin sensitivity and glucose metabolism. Receptors for ANP and BNP are abundant in human adipose tissue [26], which plays a key metabolic regulatory role. Substantially increased expression of NPR-C, the NPs clearance receptor, has been observed in the adipose tissue of hypertensive obese subjects, resulting in reduced circulating NPs levels [100,101]. An increase in NPR-C activity relative to the NPR-A signaling receptor results in a reduced NPR-A/NPR-C ratio, which makes the tissues less responsive to NPs [102]. Moreover, in obesity and insulin-resistance state, insulin may further suppress circulating NPs through upregulation of NPR-C expression [103]. Thus, adipose tissue represents a “sponge” or a “sink” for circulating NPs. On the other hand, high NPs levels are associated with reduced low-density lipoprotein (LDL) cholesterol [104], possibly due to the antagonistic effect of NPs on proprotein convertase subtilisin/kexin type 9 (PCSK9) in adipose tissue [105,106], together with increased lipolysis and energy expenditure [107,108]. NPs-dependent lipolysis can facilitate fuel availability for the heart (cardiomyocytes) and skeletal muscle during periods of increased energy needs, such as during physical activity, as fatty acids are the preferred energy substrates under such conditions [109]. As expression of visceral fat, epicardial adipose tissue is a thin to thick visceral adipose tissue layer that also penetrates the myocardium following arterial and venous epicardial vessels [110]. Thick adipose “dress” of the heart has been documented to correspond with a strong association with metabolic and CV events, especially in obese patients [111,112]. Additional data show that the NP system is a cornerstone in the crosstalk between cardiovascular and metabolic regulation [113], acting both directly on cardiac metabolism and counteracting pathological conditions, such as insulin resistance and dyslipidemia, which predispose to heart disease.

### 3.5. ANP and BNP Protect the Heart from HF

Increased ANP and BNP levels in HF are believed to be a protective hormonal response to mechanical stress in both atria and ventricles, with the goal of maintaining a healthy myocardium and CV homeostasis [114]. ANP and BNP exert crucial systemic (natriuresis/diuresis and vasodilation) and autocrine/paracrine activities in HF, compensating for the overactivation of RAAS and opposing cardiac remodeling and edema formation. Nevertheless, in the setting of chronic HF, the effectiveness of the NP system is diminished [115], despite increased secretion of ANP and BNP in both atria and ventricles. Among the various mechanisms underlying this attenuation, increased enzymatic degradation of NPs by neprilysin occurs [116], as well as an increase in NPR-C-mediated clearance [117]. In symptomatic HF, protective compensatory actions of NPs are also attenuated by enzymatic deregulation of pro-ANP/pro-BNP cleavage by corin and furin [116]. Decreased expression of these proprotein convertases leads to increased circulating levels of unprocessed NPs [118,119,120]. However, the most commonly used immunoassays for BNP, used as a prognostic and diagnostic biomarker in acute and chronic congestive HF, significantly cross react with the unprocessed pro-BNP [121]. In addition to the impairment of ANP/BNP production and degradation, symptomatic HF is characterized by NPR-A defects in ANP/BNP recognition. A diminished responsiveness to NPs has been reported in target organs, mediated by reduced NPs receptors expression, receptors desensitization and inhibited downstream signaling due to phosphodiesterases-mediated cGMP degradation [121,122]. Moreover, miR-30-GALNT1/2 axis dysregulation in HF has been found to increase the proportion of inactive pro-BNP and to impair the compensatory actions of BNP during the progression of HF [123].

Regarding HFpEF, synthesis and secretion of ANP and BNP are augmented, even during the early changes of left ventricle remodeling, when only subclinical modifications are observed [124]. This allows the NPs to be well-documented markers of hemodynamic status, and their high levels represent a hallmark of decompensated HF both with preserved and reduced ejection fraction [125]. High BP-driven concentric LV remodeling in HFpEF (compared with the most common eccentric remodeling in HFrEF) translates into reduced diastolic wall stress and thus a reduced stimulus for BNP secretion from the myocardium. Therefore, NPs circulating levels are usually lower in HFpEF compared to HFrEF for any amount of volume overload and congestion [126]. Many patients with HFpEF suffer from overweight/obesity, which is often associated with insulin-resistance, leading to increased NPs clearance and degradation [103]. Therefore, excessive adiposity and insulin resistance trigger the development of obesity-related HFpEF, even by this mechanism, also contributing to increased BP or overt hypertension and to the multiple cardiac structural modifications described above [127]. A major feature of myocardial aging strongly related to increased BP and often coexisting with HFpEF is damage of coronary microvascular circulation coupled with changes in the extracellular matrix and the development of cardiac hypertrophy and fibrosis, both in atria and ventricles [128]. The sequelae include impaired mechanical function (initially diastolic), increased risk of arrhythmias and microvascular myocardial ischemia [129]. Several studies have provided evidence that ANP and BNP play critical roles in counteracting these processes, as previously described. In a recent experimental study, Dahl salt-sensitive rats without the NPR-A for specific genetic modification, exhibited increased BP when on a high-salt diet (4% NaCl for 21 days) and intensified hypertrophy and cardiac fibrosis (with no changes in ejection fraction) compared with hypertensive Dahl rats with functional NPR-A receptors [130]. Kidney hypertrophy and increased glomerular injury scores, with reduced sodium and chloride excretion and increased activity of epithelial Na+ channel (ENaC), were also reported in these rats that could not be protected by ANP or BNP action [130]. These data clearly indicate that reduced ANP and/or BNP heart and kidney protection paves the way to HFpEF and chronic kidney disease (CKD). On the contrary, chronic ANP infusion in hypertensive Dahl rats protects from organ injury [130]. In the ARIC population, a gain-of-function polymorphism in the pro-BNP gene resulted in increased lifelong BNP levels and was found to be associated with reduced body mass index (BMI), BP and risk of incident CV disease [131]. NPs might also play a non-negligible role also in preventing coronary artery disease. In a multicenter study conducted in China, an SNP in the NPR-C gene was strongly associated with coronary atherosclerosis, although the mechanisms involved remain unclear [132].

In addition to the direct antihypertrophic, antifibrotic, antiarrhythmic and metabolic actions previously described, NPs exert several systemic actions that counteract the typical congestion of HF patients, such as increased renal perfusion and natriuresis, suppression of salt-water retention, arterial and venous dilation and inhibition of the RAAS and sympathetic nervous system [11]. The increased production and secretion of NPs in response to increased myocardial wall stress with the aim of defending the heart itself make NPs fundamental biomarkers for prognosis, detecting progression toward HF in patients with and without CV risk factors. A large Japanese population (20 to 89 years of age) without CV risk factors or clinical CV disease was studied with speckle-tracking echocardiography to unmask subclinical heart dysfunction [133]. Alterations in the left atrial (LA) reservoir and conduit function were the earliest signs of myocardial dysfunction with aging. This atrial impairment was significantly associated with an increase in BNP, independently of ventricular function. In a wide cohort of Olmsted County (Minnesota, USA) including individuals with CV risk factors or structural heart disease but without HF, elevated NT-proBNP predicted future death, HF development, myocardial infarction and stroke [134]. Elevated mid-regional pro-ANP (MR-proANP) plasma levels also predicted increased risk of major adverse CV events (MACE) and all-cause mortality in patients with type 2 diabetes mellitus, independently of CV risk factors and markers of subclinical organ damage [135]. In a case–control study, the presence of LV myocardial stiffness, representing a transitional state toward HFpEF, was nearly 30% higher in cases with LVH and elevated NT-proBNP compared to healthy controls [124].

Several clinical trials have elucidated the therapeutic role and clinical advantages of facilitating NPs activities across the HF phenotype spectrum. For example, the PARAMOUNT study on HFpEF patients showed that sacubitril/valsartan significantly reduced NT-proBNP levels compared with valsartan [136]. In the PARAGON-HF trial, which compared the effects of ARNI versus angiotensin receptor blocker (ARB) alone in patients with HFpEF, no statistically significant reduction in hospitalization and mortality for HF in the sacubitril/valsartan group was achieved in the overall population, likely due to deficiencies in the study design, although benefits have been found in some subgroups, such as patients with EF < 55% or with impaired renal function [137,138]. The PARAMETER study, which compared treatment with ARNI versus an ARB (olmesartan) in older hypertensive patients, demonstrated a significant decrease in central systolic BP and central pulse pressure in the group of patients treated with sacubitril/valsartan, providing novel insights into the potential use of ARNI for the treatment of hypertension and contrasting the progression from higher central BP to HFpEF [139]. The PARADIGM-HF trial compared the efficacy of ARNI vs. enalapril in 8442 patients with HFrEF and NYHA (New York Heart Association) classes II to IV, showing a reduction in the risk of CV death and hospitalization for HF by 20% in the sacubitril/valsartan group [52]. With administration of sacubitril/valsartan, the increased ANP in the circulation was largely more evident than the BNP increase, suggesting that the beneficial effects of neprilysin inhibition may be more influenced by ANP increase than BNP increase [53]. These multiple clinical lines of evidence clearly indicate that the NPs system works as a major counter regulator of the various mechanisms that lead to HF onset and progression [140,141].

## 4. CNP and NPR-B-Mediated Effects on the Heart

Beyond ANP and BNP, another member of the NPs family, CNP, exerts positive effects on CV health, working to maintain CV and metabolic homeostasis. CNP is mainly produced by vessel wall cells (constitutively by endothelial cells) [142]. It is also produced and released by cardiomyocytes, although its expression is likely low under basal conditions but significantly increased in HF [143]. CNP has limited natriuretic properties because it does not have the C-terminal extension after the disulfide bridge and does not bind NPR-A [16]; its effects are mainly mediated by NPR-B and through a non-cGMP-mediated pathway involving NPR-C [144]. CNP is secreted in response to several stimuli, such as hypoxia, shear stress and inflammatory cytokines [30]. Its effects involve contrasting of cardiac fibrosis and hypertrophy [145], inhibition of mesangial cell proliferation in the renal glomeruli [146] and regulation of vascular tone and BP, mainly acting on pericytes [147]. In CNP knockout mice subjected to pressure overload by abdominal aortic constriction, a series of detrimental cardiac changes were observed, such as LV dilatation, a reduction in ejection fraction and increased hypertrophy and fibrosis. The same structural and functional alterations resulted in NPR-C knockout mice, suggesting that this receptor is actively implicated in the beneficial effects mediated by CNP [148]. Moreover, a recent study suggested a role of CNP/NPR-C pathway enhancement in improving HF [149], as discussed below. In CNP knockout mice, loss of CNP signaling unexpectedly resulted in reduced body weight, diminished accumulation of adipose tissue and increased body temperature [150]. Observations in isolated murine and human adipocytes showed that CNP induces adipogenesis through the NPR-B/PKG pathway, whereas CNP/NPR-C signaling plays a crucial role in dampening sympathetic thermogenic programming. Whereas these CV and metabolic data have yet to be confirmed, CNP is thought to be involved in endochondral ossification in vivo, stimulating NPR-B both in animal models and humans. NPR-B gene gain-of-function variations and increased CNP activity by NPR-C loss lead to enhanced CNP/NPR-B activity, resulting in marked skeletal overgrowth accompanied by increased endochondral ossification and related skeletal changes, although they appear not to be correlated with relevant CV changes in vivo [151,152,153,154].

## 5. Natriuretic Peptides Clearance Receptor (NPR-C) and Musclin/Osteocrin in Cardiovascular Health

The NPs clearance receptor plays a key role in reducing the circulating levels of both ANP and BNP, thus mitigating their cellular and systemic effects [152]. The identification of some families with biallelic loss-of-function mutations in the NPR-C gene showed what happens to humans without functional NPR-C [155]. Primarily, enhanced growth and connective tissue abnormalities were observed, as evidenced by very tall stature and slender build, very long fingers, generalized joint hyperlaxity and aortic dilatation. All these features are explained by excessive activation of NPR-B by CNP on mesenchymal tissues and bones due to the lack of dampening action of NPR-C, similar to rodent animal models lacking NPR-C [152]. These children also had systolic BP below 100 mmHg and were very slender, without any sign of adiposity, suggesting an overactivation of the cardiac NPs/NPR-A pathway in adipose, adrenal and vascular tissues, with multiple predictable effects (increased adipocyte browning and heat production, decreased renin and aldosterone release, decreased tubular sodium reabsorption and direct vasodilation). The reduced ratios of NT-proBNP/BNP and NT-proANP/ANP coupled with increased cGMP plasma levels in these subjects support these assumptions [155].

**Figure 1:**
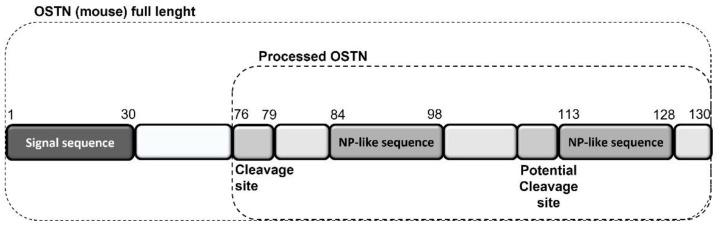
Mouse musclin/OSTN structure and NP-like sequences.

Regarding the heart, as reported above, chronic HF is associated with impaired NPs signaling, a sort of real “NPs resistance”, despite the increased production and secretion of NPs by the heart itself as a protective hormonal response [114]. NPR-C might play a central role in NP resistance as a result of enhanced internalization and degradation of NPs via NPR-C, as observed in chronic HF patients, with a consequent reduction in the biological activity of cardiac NPs [117]. In this context, musclin, also called osteocrin (OSTN) because it was originally identified as a secretory peptide from muscle and bone [156,157], has become increasingly relevant in recent years [149]. The preprocessed mature form of mouse OSTN consists of 130 amino acids. Its carboxy terminus contains tandem NP-like sequences separated by polybasic amino acids that are presumably cleaved by peptidases (Figure 1) [158].

Therefore, musclin can be considered a member of the NPs family without the “ring”, the common feature of ANP, BNP and CNP, and is mainly produced by skeletal muscles and osteoblasts [156,157]. It was also found to be expressed in the cardiomyocytes of animal models, and it is regulated by several conditions and molecules, such as physical activity, nutritional changes and fasting, hormones (especially insulin) and adiposity [156,159,160], although no direct evidence is currently available concerning the human heart. The main recent discovery is that this peptide binds NPR-C competitively and efficaciously inhibits NPR-C-mediated NPs degradation, therefore increasing NPs levels, with a consequent reduction in BP and enhanced protective activities in many tissues, including the heart [158]. Unlike humans, where the clearance action of NPR-C likely plays the most important role [102], loss of function in NPR-C mediated by musclin results in a variety of BP behaviors in experimental studies linked to the vasorelaxant activity of the CNP–NPR-C signaling pathway mainly observed in animal models. For example, Li et al. showed that musclin induced NPR-C mediated vasoconstriction and consequently increased BP in spontaneously hypertensive rats [30,161]. In skeletal muscle, musclin improves insulin-dependent glucose metabolism and enhances physical endurance by promoting mitochondrial biogenesis through NPs-induced cGMP production as a result of NPR-C blockade [160,162]. Musclin treatment prevents the worsening of congestive HF after myocardial infarction [158] and doxorubicin-induced cardiotoxicity [163] in animal models. Musclin, by binding NPR-C and increasing ANP and CNP concentrations, leads to a consequent reduction in heart and lung weights, as well as reduced cardiac fibrosis [158]. Moreover, a recently published study showed that skeletal-muscle-derived musclin protects the heart during pathological overload [149]. Musclin was found to attenuate cardiac dysfunction and myocardial fibrosis by augmenting the CNP/NPR-B-stimulated crosstalk of cGMP and cAMP in cardiomyocytes and by inhibiting p38 MAP kinase (MAPK) signaling through the activation of PKG in cardiac fibroblasts. Furthermore, musclin mRNA levels in skeletal muscle were increased by physical activity and, on the contrary, markedly downregulated in biopsies from patients suffering from HF with sarcopenia or cachexia [149]. This evidence suggests that musclin could act as a possible bridge between sarcopenia and cachexia, which are highly prevalent in advanced states of HF [164], as well as the progression of HF itself. It could represent the biological basis of the vicious circle between reduced physical activity, reduced muscle mass and HF in older patients. Overall, these important recent findings highlight the critical role of NPR-C and its interaction with musclin in reducing the protecting activities of NPs in HF patients, suggesting possible new clinical therapeutic targets for this condition.

## 6. Conclusions and Perspectives

**Figure 2:**
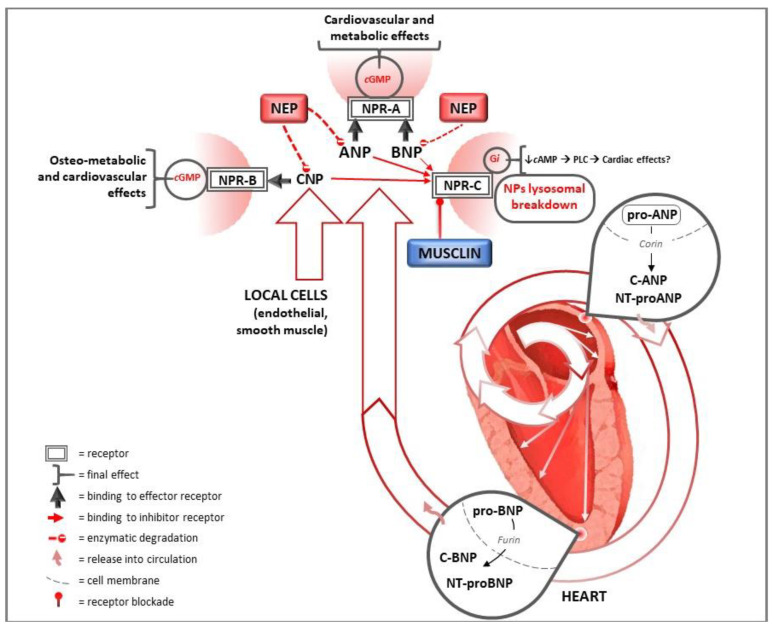
Natriuretic peptides pathways for heart and cardiovascular protection. ANP and BNP are synthesized in response to wall hemodynamic stress as precursor proteins in the heart and subsequently released into circulation after cleavage in their active form. CNP is mainly produced by endothelial cells. They exert several cardiac and systemic effects on the target organs, including the heart, binding to NPR-A and NPR-B. Their degradation is mediated by binding to NPR-C and by neprilysin. In this context, musclin competitively binds NPR-C, inhibiting NPR-C-mediated NPs degradation. Nep: neprilysin; ANP: atrial natriuretic peptide; BNP: B-type natriuretic peptide; CNP: C-type natriuretic peptide; NT: N terminal; NPR-A: natriuretic peptide receptor A; NPR-B: natriuretic peptide receptor B; NPR-C: natriuretic peptide receptor C; cGMP: cyclic guanosine monophosphate; cAMP: cyclic adenosine monophosphate; Gi: inhibitory G protein; PLC: phospholipase C.

The main studies mentioned in this review, sorted as experimental studies and clinical studies, are summarized in Supplementary Tables S1 and S2, respectively. As indicated by well-established evidence, NPs exert multiple beneficial activities on the heart and CV system (Figure 2). In this context, their paracrine and autocrine activities significantly affect heart structure and function. The development of new molecules enhancing the activities of NPs on the heart and CV system, not only through the combined inhibition of neprilysin and the AT1 angiotensin receptor but also through direct activation of the NPs system or NPR-C blockade, could lead to major advances in the treatment of heart and CV diseases, significantly reducing the major causes of morbidity and mortality worldwide.

## Data Availability

Not applicable.

## Supplementary Materials

The supporting information can be downloaded at: https://www.mdpi.com/article/10.3390/ijms232214415/s1.
