# Supplementary material for: Role of Cardiac Natriuretic Peptides in Heart Structure and Function
Source: Int J Mol Sci. 2022 Nov 20;23(22):14415. doi: 10.3390/ijms232214415 (PMC9697447; doi:10.3390/ijms232214415)
Supplement: Supplementary file 1 [file ijms-23-14415-s001.zip › ijms-1906544-supplementary.pdf]

International Journal of
Molecular Sciences

MDPI

Supplementary Materials

Role of Cardiac Natriuretic Peptides in Heart Structure and Function

Riccardo Sarzani [1,2,*], Massimiliano Allevi [1,2], Chiara Di Pentima [1], Paola Schiavi [1,2], Francesco Spannella [1,2] and Federico Giulietti [1]

[1] Internal Medicine and Geriatrics, Istituto di Ricovero e Cura a Carattere Scientifico - Istituto Nazionale di Ricovero e Cura per Anziani (IRCCS INRCA), 60127 Ancona, Italy; m.allevi@inrca.it (M.A.);
c.dipentima@inrca.it (C.D.P.); p.schiavi@inrca.it (P.S.); f.spannella@univpm.it (F.S.); f.giulietti@inrca.it (F.G.)

[2] Department of Clinical and Molecular Sciences, Università Politecnica delle Marche, 60126 Ancona, Italy

* Correspondence: r.sarzani@univpm.it (R.S.); Tel.: +39-071-5964696

Table S1. ANIMAL MODELS AND EXPERIMENTAL STUDIES REPORTED IN THE ARTICLE.

AUTHORS	STUDY POPULATION	CONTROLS	OBSERVED OUTCOMES
John et al. [1]	ANP knockout mice	Wild-type mice	Salt-sensitive hypertension
Melo et al. [2]	ANP knockout mice	Wild-type mice	Salt-sensitive hypertension
Tamura et al. [3]	BNP knockout mice	Wild-type mice	Increased cardiac fibrosis
Ogawa et al. [4]	Transgenic mice with BNP overexpression	Wild-type mice	Lower BP
Holditch et al. [5]	BNP knockout Dahl salt-sensitive rat	Dahl salt-sensitive rat without BNP deletion	Adult-onset hypertension and LVH; significant elongation of QT interval
Patel et al. [6]	Transgenic mice without NPR-A expression in cardiomyocytes	Wild-type mice	LVH and increased fibrosis after pressure overload
Otani et al. [7]	NPR-A knockout mice	Wild-type mice	LVH and fibrosis (peripartum cardiomyopathy-like remodelling)
Clements et al. [8]	Rats with induced pulmonary hypertension assigned to receive ARNI	Rats with induced pulmonary hypertension assigned to receive placebo	Reduced pulmonary pressures, vascular remodelling, and right ventricle hypertrophy
Rahmutula et al. [9]	NPR-C knockout mice	Wild-type mice	Reduction in the TGF-β1 induced atrial fibrosis
Jansen et al. [10]	NPR-C knockout mice	Wild-type mice	Exacerbated Ang II induced atrial fibrosis

Ogawa et al. [11]	BNP knockout mice	Wild-type mice	Exaggerated cardiac fibrosis
Kasahara et al. [12]	Transgenic mice overexpressing BNP in the liver and subjected to subtotal nephrectomy	Wild-type mice subjected to subtotal nephrectomy	Prevention of glomerular injury and interstitial fibrosis in the remnant kidney
Ilatovskaya et al. [13]	NPR-A knockout Dahl salt-sensitive rats on high salt diet	Hypertensive Dahl salt-sensitive rats with functional NPR-A receptors on high salt diet	Higher BP and intensified hypertrophy and cardiac fibrosis; kidney hypertrophy and higher glomerular injury scores
Moyes et al. [14]	CNP knockout mice	Wild-type mice	LV dilatation; reduction in ejection fraction; increased hypertrophy and fibrosis after pressure overload
Moyes et al. [14]	NPR-C knockout mice	Wild-type mice	LV dilatation; reduction in ejection fraction; increased hypertrophy and fibrosis after pressure overload
Szaroszyk et al. [15]	Skeletal muscle specific OSTN knockout mice	Wild-type mice	Exaggerated cardiac dysfunction and myocardial fibrosis after pressure overload
Szaroszyk et al. [15]	Transgenic mice with skeletal muscle OSTN overexpression	Wild-type mice	Attenuated cardiac dysfunction and myocardial fibrosis after pressure overload
Perez-Ternero [16]	CNP knockout mice	Wild-type mice	Reduced body weight; diminished accumulation of adipose tissue and higher body temperature
Matsukawa et al. [17]	NPR-C knockout mice	Wild-type mice	Skeletal deformities associated with increased bone turnover
Kake et al. [18]	Transgenic mice with CNP overexpression	Wild-type mice	Skeletal overgrowth without CV changes
Li et al. [19]	Spontaneously hypertensive rats	Normal rats	More marked musclin-induced vasoconstriction, reduced by blockade of NPR-C

International Journal of Molecular Sciences

Miyazaki et al. [20]	Mice subjected to continuous infusion of osteocrin after MI	Mice with MI not subjected to osteocrin infusion	Improvement of prognosis at 28 days after MI
Subbotina et al. [21]	Musclin knockout mice	Wild-type mice	Reduced exercise tolerance rescued by treatment with recombinant musclin

ANP: A-type natriuretic peptide; BNP: B-type natriuretic peptide; BP: blood pressure; LVH: left ventricular hypertrophy; NPR-A: NP receptor A; ARNI: Angiotensin Receptor-Neprilysin Inhibitor; NPR-C: NP receptor C; TGF-β1: transforming-growth-factor-beta 1; Ang II: angiotensin II; CNP: C-type natriuretic peptide; LV: left ventricular; OSTN: osteocrin; MI: myocardial infarction.

International Journal of *Molecular Sciences*

MDPI

Table S2. HUMAN CLINICAL STUDIES AND META-ANALYSES REPORTED IN THE ARTICLE.

AUTHORS	STUDY POPULATION	CONTROLS	OBSERVED OUTCOMES
Pitzalis et al. [22]	Normotensive subjects with family history of hypertension	Normotensive subjects without family history of hypertension	Specific NPR-A gene variants associated with family history of hypertension and early diastolic dysfunction
Rubattu et al. [23]	Hypertensive subjects with ANP promoter variant and NPR-A promoter variant associated with reduced ANP/NPR-A activity	Hypertensive subjects without ANP and NPR-A promoter variants	Increased LVH
Newton-Cheh et al. [24]	Individuals with the rs5068 minor G allele in the ANP gene	Individuals without the rs5068 minor G allele in the ANP gene	Increased circulating ANP levels; lower systolic and diastolic BP and reduced odds of hypertension
Cannone et al. [25]	Subjects with the rs5068 minor G allele in the ANP gene	Subjects without the rs5068 minor G allele in the ANP gene	Lower BMI, waist circumference, systolic BP, prevalence of obesity, metabolic syndrome and MI
Ellis et al. [26]	Individuals with the rs5068 minor G allele in the ANP gene	Individuals without the rs5068 minor G allele in the ANP gene	Less history of hypertension and reduced rate of CV readmission
Jujić et al. [27]	Individuals with the rs5068 minor G allele in the ANP gene	Individuals without the rs5068 minor G allele in the ANP gene	Lower likelihood of incident diabetes within 14 years
Jujić et al. [28]	Non-diabetic individuals with the rs5068 minor G allele in the ANP gene	Non-diabetic individuals without the rs5068 minor G allele in the ANP gene	Lower LV mass
McMurray et al. [29]	Patients with NYHA class II, III, or IV HF and a left ventricular ejection fraction of 40% or less assigned to receive ARNI	Patients with NYHA class II, III, or IV HF and a left ventricular ejection fraction of 40% or less assigned to receive enalapril	Lower hospitalization and mortality for HF
Wang et al. [30]	Patients with HFrEF treated with ARNI	Patients with HFrEF treated with ACE inhibitor/ARB alone	Improved LV size and hypertrophy
Spannella et al. [31]	Patients with HF treated with ARNI	Patients with HF treated with ACE inhibitor/ARB alone	Lower risk of renal dysfunction, especially

			in older patients and patients with HFpEF
Spannella et al. [32]	Patients with HFrEF treated with ARNI	Historical controls not treated with ARNI	Improvement in renal function after 12 months of treatment
Ledwidge et al. [33]	Patients with CV risk factors assigned to BNP-based screening	Patients with CV risk factors assigned to receive usual primary care	Reduction in the combined rates of LV systolic dysfunction, diastolic dysfunction, HF, and hospitalization for MACE
Scott et al. [34]	Patients with ischemic heart disease and LV dysfunction subjected to BNP measurement	-	High BNP plasma levels found to predict sudden cardiac death and ventricular arrhythmias
Garg et al. [35]	ARIC participants subjected to longitudinal NT-proBNP measurement	-	Longitudinal change in NT-proBNP associated with an increased atrial and ventricular arrhythmia burden
Hodgson-Zingman et al. [36]	Family with 11 members affected by AF	-	Heterozygous frameshift mutation in the ANP gene
Seewöster et al. [37]	Patients undergoing first AF catheter ablation	-	Prediction of LVAs by the ANP score (one point for Age ≥ 65 years, NT-proANP > 17 ng/mL, and Persistent AF)
Sarzani et al. [38]	Obese hypertensive patients with C(-55) variant in the promoter of NPR-C gene	Obese hypertensive patients without C(-55) variant	Lower circulating ANP levels and higher BP
Wang et al. [39]	Obese individuals from Framingham Study participants	Lean individuals from Framingham Study participants	Lower circulating NPs levels
Dessì-Fulgheri et al. [40]	Obese hypertensive subjects	Obese normotensive subjects	Lower NPR-A: NPR-C mRNA ratio
Spannella et al. [41]	Very elderly patients hospitalized for medical conditions	-	Negative correlations between NT-proBNP and both total cholesterol and LDL cholesterol
Hieda et al. [42]	Patients with LVH and elevated NT-proBNP	Age- and sex-matched healthy controls	Greater LV myocardial stiffness

Maisel et al. [43]	Patients with congestive HF and left ventricular ejection fraction > 45%	Patients with congestive HF and left ventricular ejection fraction < 45%	Lower circulating BNP levels
Seidelmann et al. [44]	Subjects with the rs198389 G allele in the promoter of BNP gene	Subjects with the rs198389 A allele in the promoter of BNP gene	Higher levels of NT-proBNP; reduced BP, hypertension and CV mortality, and increased lifespan
Hu et al. [45]	Subjects from Chinese Han population with NPR-C gene SNPs (rs700926, rs1833529, rs2270915, rs17541471, rs3792758 and rs696831)	Subjects from Chinese Han population without NPR-C gene SNPs	Association with increased susceptibility to coronary artery disease
Yoshida et al. [46]	Normal weight healthy participants studied with speckle-tracking echocardiography	-	Significant association between elevated BNP levels and decreases in LA reservoir and conduit strain as the earliest markers of age-related cardiac remodelling
McKie et al. [47]	Patients with stage A/B HF	Healthy normal subjects	Association between NT-proBNP values and increased risk of death, HF, cerebrovascular accident, and MI
Gauffin et al. [48]	Patients with T2DM subjected to MR-proANP measurement	-	Elevated MR-proANP levels found to predict an increased risk for MACE and all-cause mortality
Solomon et al. [49]	Patients with NYHA class II-III HF, left ventricular ejection fraction 45% or higher assigned to receive ARNI	Patients with NYHA class II-III HF, left ventricular ejection fraction 45% or higher assigned to receive ARB alone	Significant reduction of NT-proBNP levels at 12 weeks
Solomon et al. [50]	Patients with NYHA class II-III HF, left ventricular ejection fraction 45% or higher assigned to receive ARNI	Patients with NYHA class II-III HF, left ventricular ejection fraction 45% or higher assigned to receive ARB alone	No statistically significant reduction in hospitalization and mortality for HF
Williams et al. [51]	Elderly patients (aged > 60 years) with systolic	Elderly patients (aged > 60 years) with systolic	Significant reduction of systolic BP

	hypertension assigned to receive ARNI	hypertension assigned to receive ARB alone	
Szaroszyk et al. [15]	Patients with sarcopenia or cachexia due to HF	Healthy control individuals	Reduced skeletal muscle OSTN mRNA levels
Boudin et al. [52]	Individuals from three different families with bi-allelic loss-of-function mutation in NPR-C gene	-	Increased NPR-A/B signalling activity with a phenotype marked by enhanced bone growth and CV abnormalities
Andreassi et al. [53]	Patients with NYHA class I-IV HF	Age-matched healthy controls	Increased expression of NPR-C on platelets as a function of the severity of disease

NPR-A: NP receptor A; ANP: A-type natriuretic peptide; LVH: left ventricular hypertrophy; BP: blood pressure; BMI: body mass index; MI: myocardial infarction; CV: cardiovascular: LV: left ventricular; NYHA: New York Heart Association; HF: heart failure; ARNI: Angiotensin Receptor-Neprilysin Inhibitor; HFrEF: heart failure with reduced ejection fraction; ACE: angiotensin converting enzyme; ARB: angiotensin receptor blocker; HFpEF: heart failure with preserved ejection fraction; BNP: B-type natriuretic peptide; MACE: Major adverse cardiovascular events; ARIC: Atherosclerosis Risk in Communities; NT-proBNP: N-terminal pro-brain natriuretic peptide; AF: atrial fibrillation; NPR-C: NP receptor C; LDL: low-density lipoprotein; MR-proANP: Midregional pro-atrial natriuretic peptide; OSTN: osteocrin.

References

1. John, S.W.; Krege, J.H.; Oliver, P.M.; Hagaman, J.R.; Hodgin, J.B.; Pang, S.C.; Flynn, T.G.; Smithies, O. Genetic decreases in atrial natriuretic peptide and salt-sensitive hypertension. *Science* **1995**, *267*, 679–681, doi:10.1126/science.7839143.

2. Melo, L.G.; Veress, A.T.; Chong, C.K.; Pang, S.C.; Flynn, T.G.; Sonnenberg, H. Salt-sensitive hypertension in ANP knockout mice: potential role of abnormal plasma renin activity. *Am. J. Physiol.* **1998**, *274*, R255-61, doi:10.1152/ajpregu.1998.274.1.R255.

3. Tamura, N.; Ogawa, Y.; Chusho, H.; Nakamura, K.; Nakao, K.; Suda, M.; Kasahara, M.; Hashimoto, R.; Katsuura, G.; Mukoyama, M.; et al. Cardiac fibrosis in mice lacking brain natriuretic peptide. *Proc. Natl. Acad. Sci. U. S. A.* **2000**, *97*, 4239–4244, doi:10.1073/pnas.070371497.

4. Ogawa, Y.; Itoh, H.; Tamura, N.; Suga, S.; Yoshimasa, T.; Uehira, M.; Matsuda, S.; Shiono, S.; Nishimoto, H.; Nakao, K. Molecular cloning of the complementary DNA and gene that encode mouse brain natriuretic peptide and generation of transgenic mice that overexpress the brain natriuretic peptide gene. *J. Clin. Invest.* **1994**, *93*, 1911–1921, doi:10.1172/JCI117182.

5. Holditch, S.J.; Schreiber, C.A.; Nini, R.; Tonne, J.M.; Peng, K.-W.; Geurts, A.; Jacob, H.J.; Burnett, J.C.; Cataliotti, A.; Ikeda, Y. B-Type Natriuretic Peptide Deletion Leads to Progressive Hypertension, Associated Organ Damage, and Reduced Survival: Novel Model for Human Hypertension. *Hypertens. (Dallas, Tex. 1979)* **2015**, *66*, 199–210, doi:10.1161/HYPERTENSIONAHA.115.05610.

6. Patel, J.B.; Valencik, M.L.; Pritchett, A.M.; Burnett, J.C.J.; McDonald, J.A.; Redfield, M.M. Cardiac-specific attenuation of natriuretic peptide A receptor activity accentuates adverse cardiac remodeling and mortality in response to pressure overload. *Am. J. Physiol. Heart Circ. Physiol.* **2005**, *289*, H777-84, doi:10.1152/ajpheart.00117.2005.

7. Otani, K.; Tokudome, T.; Kamiya, C.A.; Mao, Y.; Nishimura, H.; Hasegawa, T.; Arai, Y.; Kaneko, M.; Shioi, G.; Ishida, J.; et al. Deficiency of Cardiac Natriuretic Peptide Signaling Promotes Peripartum Cardiomyopathy-Like Remodeling in the Mouse Heart. *Circulation* **2020**, *141*, 571–588, doi:10.1161/CIRCULATIONAHA.119.039761.

8. Clements, R.T.; Vang, A.; Fernandez-Nicolas, A.; Kue, N.R.; Mancini, T.J.; Morrison, A.R.; Mallem, K.; McCullough, D.J.; Choudhary, G. Treatment of Pulmonary Hypertension With Angiotensin II Receptor Blocker and Neprilysin Inhibitor Sacubitril/Valsartan. *Circ. Heart Fail.* **2019**, *12*, e005819, doi:10.1161/CIRCHEARTFAILURE.119.005819.

9. Rahmutula, D.; Zhang, H.; Wilson, E.E.; Olgin, J.E. Absence of natriuretic peptide clearance receptor attenuates TGF-β1-induced selective atrial fibrosis and atrial fibrillation. *Cardiovasc. Res.* **2019**, *115*, 357–372, doi:10.1093/cvr/cvy224.

10. Jansen, H.J.; Mackasey, M.; Moghtadaei, M.; Liu, Y.; Kaur, J.; Egom, E.E.; Tuomi, J.M.; Rafferty, S.A.; Kirkby, A.W.; Rose, R.A. NPR-C (Natriuretic Peptide Receptor-C) Modulates the Progression of Angiotensin II-Mediated Atrial Fibrillation and Atrial Remodeling in Mice. *Circ. Arrhythm. Electrophysiol.* **2019**, *12*, e006863, doi:10.1161/CIRCEP.118.006863.

11. Ogawa, Y.; Tamura, N.; Chusho, H.; Nakao, K. Brain natriuretic peptide appears to act locally as an antifibrotic factor in the heart. *Can. J. Physiol. Pharmacol.* **2001**, *79*, 723–729.

12. Kasahara, M.; Mukoyama, M.; Sugawara, A.; Makino, H.; Suganami, T.; Ogawa, Y.; Nakagawa, M.; Yahata, K.; Goto, M.; Ishibashi, R.; et al. Ameliorated glomerular injury in mice overexpressing brain natriuretic peptide with renal ablation. *J. Am. Soc. Nephrol.* **2000**, *11*, 1691–1701, doi:10.1681/ASN.V1191691.

13. Ilatovskaya, D. V; Levchenko, V.; Winsor, K.; Blass, G.R.; Spires, D.R.; Sarsenova, E.; Polina, I.; Zietara, A.; Paterson, M.; Kriegel, A.J.; et al. Effects of elevation of ANP and its deficiency on cardiorenal function. *JCI insight* **2022**, *7*, doi:10.1172/jci.insight.148682.

14. Moyes, A.J.; Chu, S.M.; Aubdool, A.A.; Dukinfield, M.S.; Margulies, K.B.; Bedi, K.C.; Hodivala-Dilke, K.; Baliga, R.S.; Hobbs, A.J. C-type natriuretic peptide co-ordinates cardiac structure and function. *Eur. Heart J.* **2020**, *41*, 1006–1020, doi:10.1093/eurheartj/ehz093.

15. Szaroszyk, M.; Kattih, B.; Martin-Garrido, A.; Trogisch, F.A.; Dittrich, G.M.; Grund, A.; Abouissa, A.; Derlin, K.; Meier, M.; Holler, T.; et al. Skeletal muscle derived Musclin protects the heart during pathological overload. *Nat. Commun.* **2022**, *13*, 149, doi:10.1038/s41467-021-27634-5.

16. Perez-Ternero, C.; Aubdool, A.A.; Makwana, R.; Sanger, G.J.; Stimson, R.H.; Chan, L.F.; Moyes, A.J.; Hobbs, A.J. C-type natriuretic peptide is a pivotal regulator of metabolic homeostasis. *Proc. Natl. Acad. Sci. U. S. A.* **2022**, *119*, e2116470119, doi:10.1073/pnas.2116470119.

17. Matsukawa, N.; Grzesik, W.J.; Takahashi, N.; Pandey, K.N.; Pang, S.; Yamauchi, M.; Smithies, O. The natriuretic peptide clearance receptor locally modulates the physiological effects of the natriuretic peptide system. *Proc. Natl. Acad. Sci. U. S. A.* **1999**, *96*, 7403–7408, doi:10.1073/pnas.96.13.7403.

18. Kake, T.; Kitamura, H.; Adachi, Y.; Yoshioka, T.; Watanabe, T.; Matsushita, H.; Fujii, T.; Kondo, E.; Tachibe, T.; Kawase, Y.; et al. Chronically elevated plasma C-type natriuretic peptide level stimulates skeletal growth in transgenic mice. *Am. J. Physiol. Endocrinol. Metab.* **2009**, *297*, E1339-48, doi:10.1152/ajpendo.00272.2009.

19. Li, Y.-X.; Cheng, K.-C.; Asakawa, A.; Kato, I.; Sato, Y.; Amitani, H.; Kawamura, N.; Cheng, J.-T.; Inui, A. Role of musclin in the pathogenesis of hypertension in rat. *PLoS One* **2013**, *8*, e72004, doi:10.1371/journal.pone.0072004.

20. Miyazaki, T.; Otani, K.; Chiba, A.; Nishimura, H.; Tokudome, T.; Takano-Watanabe, H.; Matsuo, A.;

Ishikawa, H.; Shimamoto, K.; Fukui, H.; et al. A New Secretory Peptide of Natriuretic Peptide Family, Osteocrin, Suppresses the Progression of Congestive Heart Failure After Myocardial Infarction. *Circ. Res.* **2018**, *122*, 742–751, doi:10.1161/CIRCRESAHA.117.312624.

21. Subbotina, E.; Sierra, A.; Zhu, Z.; Gao, Z.; Koganti, S.R.K.; Reyes, S.; Stepniak, E.; Walsh, S.A.; Acevedo, M.R.; Perez-Terzic, C.M.; et al. Musclin is an activity-stimulated myokine that enhances physical endurance. *Proc. Natl. Acad. Sci. U. S. A.* **2015**, *112*, 16042–16047, doi:10.1073/pnas.1514250112.

22. Pitzalis, M.V.; Sarzani, R.; Dessì-Fulgheri, P.; Iacoviello, M.; Forleo, C.; Lucarelli, K.; Pietrucci, F.; Salvi, F.; Sorrentino, S.; Romito, R.; et al. Allelic variants of natriuretic peptide receptor genes are associated with family history of hypertension and cardiovascular phenotype. *J. Hypertens.* **2003**, *21*, 1491–1496, doi:10.1097/00004872-200308000-00012.

23. Rubattu, S.; Bigatti, G.; Evangelista, A.; Lanzani, C.; Stanzione, R.; Zagato, L.; Manunta, P.; Marchitti, S.; Venturelli, V.; Bianchi, G.; et al. Association of atrial natriuretic peptide and type a natriuretic peptide receptor gene polymorphisms with left ventricular mass in human essential hypertension. *J. Am. Coll. Cardiol.* **2006**, *48*, 499–505, doi:10.1016/j.jacc.2005.12.081.

24. Newton-Cheh, C.; Larson, M.G.; Vasan, R.S.; Levy, D.; Bloch, K.D.; Surti, A.; Guiducci, C.; Kathiresan, S.; Benjamin, E.J.; Struck, J.; et al. Association of common variants in NPPA and NPPB with circulating natriuretic peptides and blood pressure. *Nat. Genet.* **2009**, *41*, 348–353, doi:10.1038/ng.328.

25. Cannone, V.; Boerrigter, G.; Cataliotti, A.; Costello-Boerrigter, L.C.; Olson, T.M.; McKie, P.M.; Heublein, D.M.; Lahr, B.D.; Bailey, K.R.; Averna, M.; et al. A genetic variant of the atrial natriuretic peptide gene is associated with cardiometabolic protection in the general community. *J. Am. Coll. Cardiol.* **2011**, *58*, 629–636, doi:10.1016/j.jacc.2011.05.011.

26. Ellis, K.L.; Newton-Cheh, C.; Wang, T.J.; Frampton, C.M.; Doughty, R.N.; Whalley, G.A.; Ellis, C.J.; Skelton, L.; Davis, N.; Yandle, T.G.; et al. Association of genetic variation in the natriuretic peptide system with cardiovascular outcomes. *J. Mol. Cell. Cardiol.* **2011**, *50*, 695–701, doi:10.1016/j.yjmcc.2011.01.010.

27. Jujić, A.; Nilsson, P.M.; Engström, G.; Hedblad, B.; Melander, O.; Magnusson, M. Atrial natriuretic peptide and type 2 diabetes development--biomarker and genotype association study. *PLoS One* **2014**, *9*, e89201, doi:10.1371/journal.pone.0089201.

28. Jujić, A.; Leosdottir, M.; Östling, G.; Gudmundsson, P.; Nilsson, P.M.; Melander, O.; Magnusson, M. A genetic variant of the atrial natriuretic peptide gene is associated with left ventricular hypertrophy in a non-diabetic population--the Malmö preventive project study. *BMC Med. Genet.* **2013**, *14*, 64, doi:10.1186/1471-2350-14-64.

29. McMurray, J.J. V; Packer, M.; Desai, A.S.; Gong, J.; Lefkowitz, M.P.; Rizkala, A.R.; Rouleau, J.L.; Shi, V.C.; Solomon, S.D.; Swedberg, K.; et al. Angiotensin-neprilysin inhibition versus enalapril in heart failure. *N. Engl. J. Med.* **2014**, *371*, 993–1004, doi:10.1056/NEJMoa1409077.

30. Wang, Y.; Zhou, R.; Lu, C.; Chen, Q.; Xu, T.; Li, D. Effects of the Angiotensin-Receptor Neprilysin Inhibitor on Cardiac Reverse Remodeling: Meta-Analysis. *J. Am. Heart Assoc.* **2019**, *8*, e012272, doi:10.1161/JAHA.119.012272.

31. Spannella, F.; Giulietti, F.; Filipponi, A.; Sarzani, R. Effect of sacubitril/valsartan on renal function: a systematic review and meta-analysis of randomized controlled trials. *ESC Hear. Fail.* **2020**, *7*, 3487–3496, doi:10.1002/ehf2.13002.

32. Spannella, F.; Marini, M.; Giulietti, F.; Rosettani, G.; Francioni, M.; Perna, G.P.; Sarzani, R. Renal effects of Sacubitril/Valsartan in heart failure with reduced ejection fraction: a real life 1-year follow-up study. *Intern. Emerg. Med.* **2019**, *14*, 1287–1297, doi:10.1007/s11739-019-02111-6.

33. Ledwidge, M.; Gallagher, J.; Conlon, C.; Tallon, E.; O'Connell, E.; Dawkins, I.; Watson, C.; O'Hanlon, R.; Bermingham, M.; Patle, A.; et al. Natriuretic peptide-based screening and collaborative care for heart failure: the STOP-HF randomized trial. *JAMA* **2013**, *310*, 66–74, doi:10.1001/jama.2013.7588.

34. Scott, P.A.; Barry, J.; Roberts, P.R.; Morgan, J.M. Brain natriuretic peptide for the prediction of sudden cardiac death and ventricular arrhythmias: a meta-analysis. *Eur. J. Heart Fail.* **2009**, *11*, 958–966, doi:10.1093/eurjhf/hfp123.

35. Garg, P.K.; Norby, F.L.; Wang, W.; Krishnappa, D.; Soliman, E.Z.; Lutsey, P.L.; Selvin, E.; Ballantyne, C.M.; Alonso, A.; Chen, L.Y. Association of Longitudinal Changes in Cardiac Biomarkers With Atrial and Ventricular Arrhythmias (from the Atherosclerosis Risk in Communities [ARIC] Study). *Am. J. Cardiol.* **2021**, *158*, 45–52, doi:10.1016/j.amjcard.2021.07.043.

36. Hodgson-Zingman, D.M.; Karst, M.L.; Zingman, L. V; Heublein, D.M.; Darbar, D.; Herron, K.J.; Ballew, J.D.; de Andrade, M.; Burnett, J.C.J.; Olson, T.M. Atrial natriuretic peptide frameshift mutation in familial atrial fibrillation. *N. Engl. J. Med.* **2008**, *359*, 158–165, doi:10.1056/NEJMoa0706300.

37. Seewöster, T.; Büttner, P.; Zeynalova, S.; Hindricks, G.; Kornej, J. Are the atrial natriuretic peptides a missing link predicting low-voltage areas in atrial fibrillation? Introducing the novel biomarker-based atrial fibrillation substrate prediction (ANP) score. *Clin. Cardiol.* **2020**, *43*, 762–768, doi:10.1002/clc.23378.

38. Sarzani, R.; Dessì-Fulgheri, P.; Salvi, F.; Serenelli, M.; Spagnolo, D.; Cola, G.; Pupita, M.; Giantomassi, L.; Rappelli, A. A novel promoter variant of the natriuretic peptide clearance receptor gene is associated with lower atrial natriuretic peptide and higher blood pressure in obese hypertensives. *J. Hypertens.* **1999**, *17*, 1301–1305, doi:10.1097/00004872-199917090-00010.

39. Wang, T.J.; Larson, M.G.; Levy, D.; Benjamin, E.J.; Leip, E.P.; Wilson, P.W.F.; Vasan, R.S. Impact of obesity on plasma natriuretic peptide levels. *Circulation* **2004**, *109*, 594–600, doi:10.1161/01.CIR.0000112582.16683.EA.

40. Dessì-Fulgheri, P.; Sarzani, R.; Tamburrini, P.; Moraca, A.; Espinosa, E.; Cola, G.; Giantomassi, L.; Rappelli, A. Plasma atrial natriuretic peptide and natriuretic peptide receptor gene expression in adipose tissue of normotensive and hypertensive obese patients. *J. Hypertens.* **1997**, *15*, 1695–1699, doi:10.1097/00004872-199715120-00074.

41. Spannella, F.; Giulietti, F.; Cocci, G.; Landi, L.; Borioni, E.; Lombardi, F.E.; Rosettani, G.; Bernardi, B.; Bordoni, V.; Giordano, P.; et al. N-terminal pro B-Type natriuretic peptide is inversely correlated with low density lipoprotein cholesterol in the very elderly. *Nutr. Metab. Cardiovasc. Dis.* **2018**, doi:10.1016/j.numecd.2018.02.013.

42. Hieda, M.; Sarma, S.; Hearon, C.M.J.; Dias, K.A.; Martinez, J.; Samels, M.; Everding, B.; Palmer, D.; Livingston, S.; Morris, M.; et al. Increased Myocardial Stiffness in Patients With High-Risk Left Ventricular Hypertrophy: The Hallmark of Stage-B Heart Failure With Preserved Ejection Fraction. *Circulation* **2020**, *141*, 115–123, doi:10.1161/CIRCULATIONAHA.119.040332.

43. Maisel, A.S.; McCord, J.; Nowak, R.M.; Hollander, J.E.; Wu, A.H.B.; Duc, P.; Omland, T.; Storrow, A.B.; Krishnaswamy, P.; Abraham, W.T.; et al. Bedside B-Type natriuretic peptide in the emergency diagnosis of heart failure with reduced or preserved ejection fraction. Results from the Breathing Not Properly Multinational Study. *J. Am. Coll. Cardiol.* **2003**, *41*, 2010–2017, doi:10.1016/s0735-1097(03)00405-4.

44. Seidelmann, S.B.; Vardeny, O.; Claggett, B.; Yu, B.; Shah, A.M.; Ballantyne, C.M.; Selvin, E.; MacRae, C.A.; Boerwinkle, E.; Solomon, S.D. An NPPB Promoter Polymorphism Associated With Elevated N-Terminal pro-B-Type Natriuretic Peptide and Lower Blood Pressure, Hypertension, and Mortality. *J. Am. Heart Assoc.* **2017**, *6*, doi:10.1161/JAHA.116.005257.

45. Hu, Q.; Liu, Q.; Wang, S.; Zhen, X.; Zhang, Z.; Lv, R.; Jiang, G.; Ma, Z.; He, H.; Li, D.; et al. NPR-C gene polymorphism is associated with increased susceptibility to coronary artery disease in Chinese Han population: a multicenter study. *Oncotarget* **2016**, *7*, 33662–33674, doi:10.18632/oncotarget.9358.

46. Yoshida, Y.; Nakanishi, K.; Daimon, M.; Ishiwata, J.; Sawada, N.; Hirokawa, M.; Kaneko, H.; Nakao, T.; Mizuno, Y.; Morita, H.; et al. Alteration of Cardiac Performance and Serum B-Type Natriuretic Peptide Level in Healthy Aging. *J. Am. Coll. Cardiol.* **2019**, *74*, 1789–1800, doi:10.1016/j.jacc.2019.07.080.

47. McKie, P.M.; Cataliotti, A.; Lahr, B.D.; Martin, F.L.; Redfield, M.M.; Bailey, K.R.; Rodeheffer, R.J.; Burnett, J.C.J. The prognostic value of N-terminal pro-B-type natriuretic peptide for death and cardiovascular events in healthy normal and stage A/B heart failure subjects. *J. Am. Coll. Cardiol.* **2010**, *55*, 2140–2147, doi:10.1016/j.jacc.2010.01.031.

48. Gauffin, E.; Chisalita, S.I.; Engvall, J.; Nyström, F.H.; Östgren, C.J. Plasma mid-regional pro-atrial natriuretic peptide predicts cardiovascular events in patients with type 2 diabetes independently of subclinical organ damage. *Diabetes Res. Clin. Pract.* **2021**, *182*, 109095, doi:10.1016/j.diabres.2021.109095.

49. Solomon, S.D.; Zile, M.; Pieske, B.; Voors, A.; Shah, A.; Kraigher-Krainer, E.; Shi, V.; Bransford, T.; Takeuchi, M.; Gong, J.; et al. The angiotensin receptor neprilysin inhibitor LCZ696 in heart failure with preserved ejection fraction: a phase 2 double-blind randomised controlled trial. *Lancet (London, England)* **2012**, *380*, 1387–1395, doi:10.1016/S0140-6736(12)61227-6.

50. Solomon, S.D.; McMurray, J.J. V; Anand, I.S.; Ge, J.; Lam, C.S.P.; Maggioni, A.P.; Martinez, F.; Packer, M.; Pfeffer, M.A.; Pieske, B.; et al. Angiotensin-Neprilysin Inhibition in Heart Failure with Preserved Ejection Fraction. *N. Engl. J. Med.* **2019**, *381*, 1609–1620, doi:10.1056/NEJMoa1908655.

51. Williams, B.; Cockcroft, J.R.; Kario, K.; Zappe, D.H.; Brunel, P.C.; Wang, Q.; Guo, W. Effects of Sacubitril/Valsartan Versus Olmesartan on Central Hemodynamics in the Elderly With Systolic Hypertension: The PARAMETER Study. *Hypertens. (Dallas, Tex. 1979)* **2017**, *69*, 411–420, doi:10.1161/HYPERTENSIONAHA.116.08556.

52. Boudin, E.; de Jong, T.R.; Prickett, T.C.R.; Lapauw, B.; Toye, K.; Van Hoof, V.; Luyckx, I.; Verstraeten, A.; Heymans, H.S.A.; Dulfer, E.; et al. Bi-allelic Loss-of-Function Mutations in the NPR-C Receptor Result in Enhanced Growth and Connective Tissue Abnormalities. *Am. J. Hum. Genet.* **2018**, *103*, 288–295, doi:10.1016/j.ajhg.2018.06.007.

53. Andreassi, M.G.; Del Ry, S.; Palmieri, C.; Clerico, A.; Biagini, A.; Giannessi, D. Up-regulation of "clearance" receptors in patients with chronic heart failure: a possible explanation for the resistance to biological effects of cardiac natriuretic hormones. *Eur. J. Heart Fail.* **2001**, *3*, 407–414, doi:10.1016/s1388-9842(01)00161-1.
